# The Regulatory Activity of Noncoding RNAs in ILCs

**DOI:** 10.3390/cells10102742

**Published:** 2021-10-14

**Authors:** Alessio Grimaldi, Giuseppe Pietropaolo, Helena Stabile, Andrea Kosta, Cristina Capuano, Angela Gismondi, Angela Santoni, Giuseppe Sciumè, Cinzia Fionda

**Affiliations:** 1 Department of Molecular Medicine, Istituto Pasteur-Fondazione Cenci Bolognetti, Sapienza University of Rome, 00161 Rome, Italy; alessio.grimaldi@uniroma1.it (A.G.); giuseppe.pietropaolo@uniroma1.it (G.P.); helena.stabile@uniroma1.it (H.S.); andrea.kosta@uniroma1.it (A.K.); angela.gismondi@uniroma1.it (A.G.); angela.santoni@uniroma1.it (A.S.); giuseppe.sciume@uniroma1.it (G.S.); 2 Department of Experimental Medicine, Sapienza University of Rome, 00161 Rome, Italy; cristina.capuano@uniroma1.it; 3 IRCCS (Istituto di Ricovero e Cura a Carattere Scientifico) Neuromed, 86077 Pozzilli, Italy

**Keywords:** innate lymphoid cells, noncoding RNA, microRNA, long noncoding RNA, circular RNA

## Abstract

Innate lymphoid cells (ILCs) are innate lymphocytes playing essential functions in protection against microbial infections and participate in both homeostatic and pathological contexts, including tissue remodeling, cancer, and inflammatory disorders. A number of lineage-defining transcription factors concurs to establish transcriptional networks which determine the identity and the activity of the distinct ILC subsets. However, the contribution of other regulatory molecules in controlling ILC development and function is also recently emerging. In this regard, noncoding RNAs (ncRNAs) represent key elements of the complex regulatory network of ILC biology and host protection. ncRNAs mostly lack protein-coding potential, but they are endowed with a relevant regulatory activity in immune and nonimmune cells because of their ability to control chromatin structure, RNA stability, and/or protein synthesis. Herein, we summarize recent studies describing how distinct types of ncRNAs, mainly microRNAs, long ncRNAs, and circular RNAs, act in the context of ILC biology. In particular, we comment on how ncRNAs can exert key effects in ILCs by controlling gene expression in a cell- or state-specific manner and how this tunes distinct functional outputs in ILCs.

## 1. Introduction

Innate lymphoid cells (ILCs) are a heterogeneous population of innate lymphocytes, which originate from the common lymphoid progenitor but lack antigen-specific receptors [1]. Based on their phenotype and the specific expression of transcription factors (TFs) and cytokines, ILCs have been categorized into five prototypical subsets [2].

Natural killer (NK) cells and type-1 innate lymphoid cells, namely ILC1, are mainly involved in the protective immune response against viruses and intracellular bacteria as well as in cancer immunosurveillance. These subpopulations share the expression of the TF T-BET and the ability to produce interferon (IFN)-γ, but only NK cells are highly cytotoxic and require EOMES for their development [3]. Many of the phenotypic and functional properties of NK cells and ILC1 are strictly tissue dependent; however, while the border separating NK cells and ILC1 has become very thin in mice, how these two subsets unambiguously segregate in humans is still puzzling [4,5,6,7]. In this context, a unique ILC1-like subset can be generated from NK cells in distinct tissues, such as liver, salivary gland, and intestine, as well as in the tumor microenvironment by transforming growth factor-β (TGF-β) [8,9,10].

Type-2 innate lymphoid cells (ILC2) are characterized by high expression levels of the TF GATA3 [11,12] and play a key role in allergic reactions and protection against parasitic infections via the secretion of interleukin (IL)-5, IL-9, IL-13, and amphiregulin [13,14]. ILC2 are enriched in several tissues, including intestine, lung, and bone marrow and can also be found in the peripheral blood of healthy individuals, although at a very low frequency (less than 0.1% of total leucocytes) as compared to NK cells. The heterogeneity of ILC2 has been considered limited, compared to other ILC subsets. However, upon inflammation, an ILC2 subset, referred to as “inflammatory ILC2”, can acquire the ability to recirculate and to produce IL-17, both in mice and humans [15,16,17,18,19].

Type-3 innate lymphoid cells (ILC3) depend on the transcription factor RORγt and secrete high amount of IL-17 and IL-22 [20]. ILC3 are mainly localized in tonsils and intestinal lamina propria, and subsets of these cells are generally distinguished by the presence or absence of NCR receptors (NKp44 in humans and NKp46 in mice) [20,21]. ILC3 stimulate the differentiation of epithelial cells from intestinal stem cells, promote the antimicrobial response by epithelial cells, and induce neutrophil recruitment/activation [22,23]. Finally, lymphoid tissue inducer (LTi) cells regulate the formation of lymph nodes and Peyer’s patches during embryonic development, mainly through the production of lymphotoxin. The development of these cells depends on the TF RORγt, which also controls the fate of LTi-like cells present in the adult lymphoid and nonlymphoid tissues [24,25].

In roughly the last 10 years, our understanding of ILC biology has rapidly grown; however, the molecular pathways controlling development and functions of ILCs are still widely expanding. The TF EOMES, T-BET, GATA3, and RORγt, mentioned above, are also referred to as lineage defining TFs (LDTFs), since these molecules dictate ILC fates and are required for determining the effector functions of mature ILC subsets [26,27]. LDTFs represent the first layer of ILC regulation, although the establishment of specific developmental programs and effector functions is now seen as the result of complex TF networks rather than the effect of one single “master” regulator [28].

Whole-transcriptome RNA sequencing data suggest that transcription can occur across almost the entire genome, generating a myriad of RNA molecules without protein-coding functions, named noncoding RNAs (ncRNAs). ncRNAs have relevant regulatory properties and control several biological processes. ncRNAs include microRNA (miRNAs), ribosomal RNA (rRNAs), transfer RNA (tRNAs), long ncRNAs (lncRNAs), and circular RNAs (circRNAs) [29]. Some of the most widely studied classes of nc-RNAs, miRNAs, lncRNAs, and circRNAs are active in the control gene expression [30]. Moreover, several pieces of evidence showed that they are also involved in innate or adaptive immune responses [31,32,33]. Regarding ILCs, miRNAs are known regulators of NK cell biology and control their development, activation, and effector functions [34]. However, the miRNA content and regulatory function in other human ILC subsets have been poorly investigated. More recently, some studies described the functions of specific lnc- and circ-RNAs in distinct ILC subpopulations. Here, we summarize the latest research on ILC subsets related to miRNAs, lncRNAs, and circRNAs and discuss their critical roles in mechanisms underlying ILC development, activation, and function.

## 2. Regulation of ILC Activity by miRNAs

### 2.1. Properties of miRNAs

The discovery of the first miRNA in 1993 paved the way for the hypothesis that gene regulation was not only coordinated by proteins but also by RNA molecules [35,36]. The biogenesis of miRNA starts in the nucleus, where miRNAs are transcribed in primary transcripts (also known as pri-miRNAs) by RNA polymerase II and processed into long hairpin precursors of ∼70–100 nucleotides (pre-miRNAs) by Drosha [37,38]. After that, pre-miRNAs are transported to the cytoplasm where pre-miRNAs are cleaved by Dicer to form mature miRNAs [39]. This cleavage creates a double strand of ∼22-nucleotides, including a mature miRNA guide strand and a mature complementary passenger strand. Mature miRNAs are then loaded into the RNA-induced silencing complex (RISC). The recruitment of the RISC complex to the target mRNA, mediated by binding of the mature miRNA to a complementary sequence in the 3′UTR of target mRNAs, leads to mRNA degradation or translational suppression [40]. Each miRNA can regulate several mRNAs, and one single mRNA can be targeted by different miRNAs, generating a complex regulatory circuit able to control several biological processes, including differentiation, development, metabolism, proliferation, apoptosis, viral infection, tumorigenesis, and immunity [41,42].

### 2.2. miRNAs and ILCs

In 2007, the findings obtained by two independent groups by knocking out miR-155 in mice provided evidence for the central role that miRNAs can have in controlling biological processes, and, specifically, the immune system [43,44]. In this regard, miRNAs control developmental pathways and effector functions of several immune cells [45,46,47]. Integrative approaches combining comprehensive analysis of chromatin modifications, transcriptome, and miRNome of mouse developing and differentiated adaptive and immune cells have shed light on the complex mechanisms regulating miRNA-specific expression and abundance during lymphopoiesis [48]. These findings corroborated the hypothesis for a developmental regulation of miRNA strand accumulation and also showed that lymphocyte specific expression of miRNAs is obtained via epigenetic regulation, involving gene silencing through the trimethylation of lysine 27 of the histone 3 (H3K27me3). Recently, the miRNA profiles of 63 primary mouse immune populations, also including spleen and liver NK cells from healthy or cytomegalovirus infected mice, have been established within the context of the ImmGen program, unveiling the expression of both shared and unique miRNAs by each cell type. By integrating data from miRNA profiles with global DNA-accessibility, histone mark distribution, and nascent RNA profiles, it has emerged that miRNAs can use multiple promoters as a mechanism capable of maintaining specificity and abundance in each immune population, thus adding further information on the regulatory landscape of immune cells [49].

**Figure 1:**
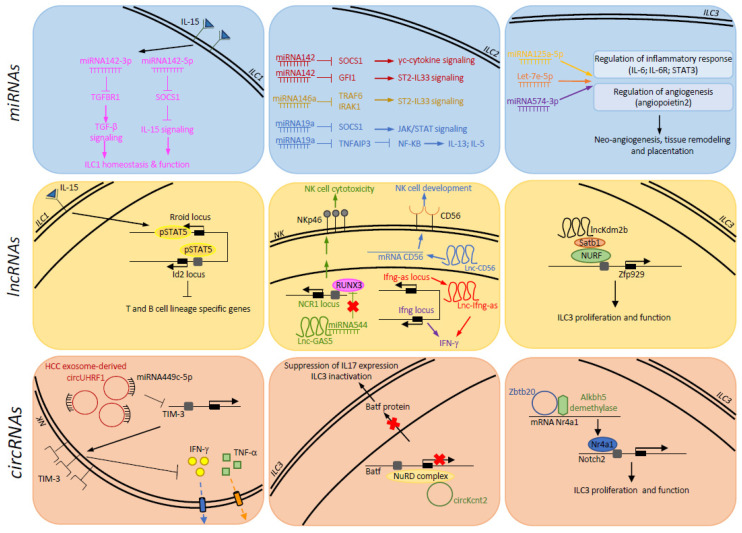
Functions of ncRNAs in ILCs. Molecular mechanisms underlying the regulatory effects of miRNAs (blue boxes), lncRNAs (yellow boxes), and circRNAs (red boxes) on the development and/or activity of distinct ILC subsets (NK, ILC1, ILC2 and ILC3). Single- and double-black lines indicate nuclear membrane and cytoplasmic membrane, respectively. Human and mouse gene names are indicated in capital and small letters, respectively. Arrow and block symbols indicate positive and negative regulation of mechanisms, respectively.

The pleiotropic role for miRNAs in NK cell biology was initially suggested by studies employing mouse models, in which the ablation of Dicer was induced by drug (Tamoxifen/CreERT2 system) or in cells expressing NKp46 (*Ncr1^iCRE^*-mediated *Dicer1* inactivation) [50,51]. Indeed, these mice were characterized by severe defects in NK cell maturation/differentiation and significant phenotypical and functional alterations, including the ability to protect against cytomegalovirus infection and cancer growth. Since then, the regulatory mechanisms underlying the impact of distinct miRNAs on the development, activation, and effector functions of mouse and human NK cells have been elucidated. The importance of miRNAs on NK cell biology has been intensively reviewed and is not discussed here (interested readers are referred to other outstanding reviews [52,53,54,55]. We focus on the role of miRNAs in the other ILC subsets (Figure 1, upper panel).

Among miRNAs, miR-142-3p/5p, encoded by *Mir142* gene, are required for the development of different hematopoietic cells, such as mast cells, dendritic cells, erythrocytes, and adaptive lymphocytes [56,57]. As well, miR-142-3p/5p play a broad role in regulation of ILC functions. These miRNA isoforms are present in high levels in mature ILC1, and their expression can be further increased by IL-15. Both germline and conditional deletion of *Mir142*, by using *Ncr1-cre* × *Mir142^fl/fl^* mice, have highlighted the importance of this miRNA in ILC1 homeostasis and function [58]. The loss of *Mir142* causes a strong reduction of ILC1 and NK cell compartments, the latter results mainly represented by ILC1-like NK cells, due to the altered activity of two crucial cytokines for NK/ILC1 homeostasis, IL-15, and TGF-β [59,60]. Indeed, while miR142-5p inhibits the expression of the negative regulator of the IL-15 signaling, *Socs1*; miR142-3p directly targets *Tgfbr1*. Consequently, in *miR142*-deficient mice, the homeostatic activity of IL-15 is compromised by the enhanced Socs1 levels, explaining the lower number of NK cells and ILC1. On the other hand, the TGF-β signaling is directly potentiated, likely inducing ILC1-like NK cells. Along with the regulation of NK cell/ILC1 homeostatic functions, mir142 exerts important regulatory functions also in the mouse ILC2 compartment. This miRNA plays a cell-intrinsic role in defining the homeostatic pool of bone marrow ILC2, and it also controls the phenotypic and functional properties of mature ILC2 at mucosal sites [61]. The absence of miR-142 results in the accumulation in ILC2 in the bone marrow, and this is independent from the effects on the earliest fully committed helper-like ILC precursor (ILCp) and α-lymphoid progenitors (αLP). In the peripheral tissues, *Mir142*^−/−^ ILC2 have enhanced the surface expression of typical ILC2 markers, including CD25, Sca-1, Klrg1, ST2 (IL-33R), and IL-25R. Even though the phenotypic features observed in *Mir142*^−/−^ ILC2 might be associated with an enhanced activation state, these cells are severely defective in their proliferative and effector responses during *N. brasiliensis* infection, as well as at baseline. While miR142 isoform expression levels could be reduced by IL-33 and IL-25, the direct miR142 targets include important regulators of the cytokine-induced pathways, such as *Socs1* and *Gfi1* [62]. As described for ILC1, the loss of miR142 enhances Socs1 expression, leading to a defective γc-cytokine signaling in ILC2. In addition, the transcription factor Gfi1 could also regulate the responsiveness of ILC2 to IL-33 by inducing the expression of its receptor ST2.

Profiling the miRNA expression of lung ILC2 showed that *Socs1* could also be targeted by another miRNA, miR19a [63]. This miRNA is part of the miRNA 17–92 cluster which plays a critical role in lung ILC2 homeostasis. Lung ILC2 lacking this cluster exhibited defective proliferation and cytokine production at a steady state and during allergic response. In addition to Socs1, the mir19a-mediated repression of *Tnaifp3*, encoding for A20, a negative regulator of NF-kB, specifically regulates IL-5 and IL-13 production. Accordingly, the depletion of Tnaifp3 and Socs1 in *Mir17-92*^−/−^ mice or miR19a transfection is sufficient to increase ILC2 cytokine production. miRNA 17-92 cluster is included within a large group of miRNAs shared between ILC2 and Th2 cells, consistently with their similar gene expression and cytokine profiles. These findings suggest that the overlapping miRNA repertoires could be used by innate and adaptive lymphocytes to generate similar effector functions; thus, we cannot exclude that this also occurs for ILC1 vs. Th1 and ILC3 vs. Th17. In this regard, several miRNAs described in Th cells could be able to target shared pathways in ILCs. For instance, miR-29 has been shown to be essential for suppression of Th1 differentiation and for limiting NK cell functions by directly targeting the LDTFs *T-bet* and *Eomes* and the type 1 signature cytokine, IFN-γ [64,65]. In addition, miR-221 and miR-222 are able to limit generation of pathogenic Th17 by targeting *Maf* and *Il23r* [66]. Whether and how these and other miRNAs could regulate ILCs remain to be addressed.

As mentioned above, miR155 plays a pivotal role in regulating the functions of immune cells, and emphasis has been given to its role on the adaptive branch of the immunity. However, miR155 also represents a critical regulator of ILC2 and NK cell biology, impacting development and functions [67,68]. In the context of ILC2, a number of studies focused on mouse models of allergic airway inflammation demonstrated a strong impact of this miRNA on these lymphocytes via the alteration of IL-33 signaling required for their proliferation and function as well as via direct changes of their gene expression [11,69]. *Mir155*-deficient mice are protected against the allergic inflammation because of a lower number of neutrophils, lymphocytes, eosinophils, and ILC2 in the lung. Importantly, the lack of miR155 negatively affects IL-33 signaling causing reduced IL-33 production and increased expression of its receptor ST2. However, IL-33 is not sufficient to increase ILC2 numbers in *miR155*^−/−^ mice and to enhance IL-13 production and GATA3 expression or proliferation of *Mir155*^−/−^ ILC2s. These findings highlight the relevance of a cell-intrinsic role of miR155 in ILC2, and IL-33-induced miR155 may regulate cytokine secretion and the expansion of ILC2. Among miR155 targets, an important role is also assumed for c-Maf, a TF known to suppress IL-4, IL-5, IL-9, and IL-13 production [70]. Like miR155, a member of the miR146 family, miR146a controls IL-33/ST2 pathway in mouse ILC2. Treatment of ILC2 with IL-33 results in the enhanced levels of miR146a, which inhibits the expression of TRAF6 and IRAK1, two key proteins of IL-33/ST2 signaling. Consistently, miR146a inhibits ILC2 proliferation and function [71].

The role of miRNAs in the regulation of ILC3 biology remains poorly investigated, and current evidence is restricted to ILC3 isolated from the human decidua and tonsils [72]. Like NK cells, decidual ILC3 (dILC3) regulate the implantation and maintenance of pregnancy because of their role in neoangiogenesis, tissue remodeling, and placentation [73]. A comprehensive miRNA expression analysis of NCR^+^ dILC3 isolated during the first trimester of pregnancy revealed a unique miRNA profile for these cells, compared with those of decidual (dNK) and peripheral blood NK cells (pbNK) [74]. In particular, the miR-125a-5p, let-7e-5p, and miR-574-3p resulted as highly expressed in dILC3. These miRNAs can potentially regulate genes involved in different biological processes (e.g., innate immune response, cytokine production, and tissue remodeling), sharing target genes implicated in the regulation of inflammatory response (e.g., IL6, IL6R, and STAT3), and angiogenesis (e.g., angiopoietin 2). These findings suggest that, during the early phases of pregnancy, the regulation of gene expression by these miRNAs contribute to limiting the excessive response of dILC3 that could compromise implantation and tissue remodeling. Tonsil ILC3 also express high levels of miR-125a-5p, let-7e-5p, and miR-574-3p suggesting a role for these miRNAs in defining the identity and functions of this population. Profiling the miRNome in distinct ILC3 subsets from different tissues is helpful for addressing this possibility.

## 3. Regulation of ILC Activity by lncRNAs

### 3.1. Properties of lncRNAs

LncRNAs are classified as long RNA transcripts with more than 200 nucleotides. This cutoff of 200 nucleotides helps to discriminate lncRNAs from the classes of small RNA, such as transfer RNA and miRNAs. The estimated number of lncRNA loci ranges from 10,000 to over 100,000 in the human genome (ENCODE Project Consortium, 2012), and compared to other RNAs, lncRNAs are less conserved and have lower expression levels [75]. Although most lncRNAs are transcribed by RNA Polymerase II and share several processes with mRNA biogenesis (capping, polyadenylation, and splicing), their transcription, processing, and export occur through distinct mechanisms which are strongly connected with cellular fate, localization, and function [76]. The lncRNA map in different genomic locations and based on their position relative to gene loci encoding protein-coding mRNA can be found as intronic sequences, antisense regions, within coding genes, or as bidirectional and intergenic regions. LncRNAs can act close to their site of transcription (in cis) or at distant locations (in trans) by several molecular mechanisms functioning as signal, decoy, guide, or scaffold molecules [77]. These transcription products play a crucial role in the fine-tuning of nuclear organization, RNA processing, transcriptional and post-transcriptional machinery and in the modulation of crucial functions of other ncRNAs [78].

### 3.2. LncRNAs and ILCs

While the number of lncRNAs identified in immune cells is growing, our knowledge of the impact of these molecules on immune cells [79,80] and, in particular, on ILCs is still limited (Figure 1, middle panel). The whole-genome RNA-seq profiling of thymocytes, mature T cells and distinct Th cell populations in humans and mice has led to the identification of thousands of genomic regions able to generate lncRNAs, which are generally adjacent to and co-expressed with, protein-coding genes regulating immune functions [81,82]. This evidence implies a role of lncRNAs in the regulation of T-cell development and polarization. In addition, distinct LDTFs, namely T-BET and GATA3, as well as STATs, can drive Th1/Th2 specific expression of lncRNAs [81]. Among the Th-specific lncRNAs, a cluster comprising four alternatively spliced lncRNAs is selectively expressed on Th2, and it is able to regulate the expression of type 2 cytokines [82]. This lncRNA cluster overlaps the *RAD50* gene in humans, which is located between the *Il13* and *Il5* loci, and is contiguous with the Th2 locus control region (LCR) described in mice, and for this reason, it has been designated as Th2-LCR lncRNA. This aspect is highly relevant in the context of ILC2 regulation of gene expression, since Th2 and ILC2 undergo a substantial convergence of their regulomes during infection and the DNA accessibility profile of the type 2 locus highly overlaps the two populations [83]. As an example of common mechanism of regulation in diverse immune cells, Ifng-as (also referred as NeST and Tmevpg1) controls *Ifng* expression in T lymphocytes and NK cells [84]. By using a genetic approach targeting either the entire locus or only Ifng-as1 RNA transcription, it has been observed that a double mechanism involving both the Ifn-as1 DNA locus and its transcript is necessary for the optimal expression of *Ifng*. In particular, the Ifn-as1 locus is an important cisregulatory element for *Ifng* required for proper remodeling of the chromatin structure. In addition, Ifng-as1 RNA serves to promote the binding of transcription factors and/or chromatin modifiers, but it can also exert effects on mRNA stability. Interestingly, the induction of Ifng-as1 expression is dependent upon Stat4 and T-bet transcription factors, also required for *Ifng* transcription. Despite these molecular events having been dissected in T cells, NK cells from Ifng-as1 deficient mice also produce reduced levels of IFN-γ. Moreover, the regulatory function of this lncRNA seems preserved in human NK cells where the overexpression of IFN-AS1 enhances IFN-γ secretion [85], and the amount of this lncRNA is significantly increased by activating cytokines, such as IL-12 alone or in combination with IL18.

Based on the specific transcriptional programs underlying the specification of ILC fates, it is plausible that ILC identity is also defined by the expression of precise sets of lncRNAs. The comparison of global lncRNA expression in human pbNK, cord blood (cbNK), and dNK cells has revealed NK-lncRNA signatures consisting of 1632 lncRNAs [86]. Most of these lncRNAs are coexpressed among the different human NK cell populations; however, pbNK and cbNK cells share more lncRNAs with each other, with respect to dNK cells. Among the shared lncRNAs, the possible involvement in the regulation of NK cell biology has been hypothesized for lncRNA AK096651 and AB128931 (also named lncCD56), based on their predicted targets. Indeed, AK096651 putative targets include CD160, a receptor triggering NK-mediated IFN-γ production, which defines ILC1 populations able to provide potent IFN-γ responses both in the intestinal epithelium and liver [87,88]. On the other hand, lnc-CD56 has been predicted to interact with the TFs *TBX21*, *IRF2, IKZF2, ELF4,* and *EOMES* and to target *CD56*, a classical human NK cell surface marker. The regulation of CD56 has been validated by in vitro studies showing that the silencing of lncCD56 significantly reduces the surface expression of CD56 on dNK cells. As an adhesion molecule, CD56 regulates contact-dependent processes between developing NK cells and stromal cells [89]. Accordingly, the knockdown of lncCD56 also compromises the differentiation of NK cells from CD34^+^ hematopoietic progenitor cells.

The possibility that lncRNAs contribute to determining phenotypes and functions of NK cells derived from different cell compartments is also supported by evidence on the changes in the lncRNA expression pattern among diverse cell states and in pathologic conditions. Accordingly, 67 lncRNAs were found specifically expressed in dNK cells isolated from patients with early nonchromosome-related missed abortion (MA) but not in healthy controls [90]. The dysregulated expression of these lncRNAs was associated with defects in IL-1- and IL-15-mediated signaling and the phosphatidylinositol signaling system, but also in pathways regulating cell adhesion and metabolism. Thus, a specific profile of lncRNAs may account for dNK cell abnormalities in the case of MA, suggesting that further investigation of the role of these lncRNAs in NK and other ILC populations would improve our knowledge on the regulatory circuits underpinning their activity in a variety of disease conditions, including inflammation and cancer. To this regard, pbNK cells from patients with liver cancer can express reduced levels of the lncRNA GAS5, and this correlates with NK cell dysfunctions and worse patients’ prognoses [91]. The lncRNA GAS5 expression was elevated in IL-2 activated-NK cells and serves as a positive regulator of NK cell functions through indirect regulation of the activating receptor *NCR1*/NKp46. The lncRNA GAS5 is a decoy for miR544 and blocks its activity. In particular, the binding of the lncRNA GAS5 to miR-544 prevents the repression of RUNX3, a relevant transcriptional activator of the *NCR1* gene. The upregulation of NKp46 expression leads to enhanced NK cell cytokine production and cytotoxicity.

Regulatory functions of lncRNAs have been also described in ILC1 and ILC3. Mowel and colleagues identified the lncRNA Rroid as being specifically expressed in NK cells and ILC1 but not in other ILC subsets [92]. Mice deficient of the Rroid locus (*Rroid*^−/−^) display decreased frequency and number of NK cells and ILC1 in most tissues including spleen, liver, lung, and intestine but comparable amounts of intestinal and lung ILC2 and ILC3, compared with wild-type mice. The reduction of NK cells and ILC1 is dependent on a defective expression of Id2, a negative regulator of the E-protein TFs, which are responsible for the activation of T- and B-cell lineage-specific genes [93,94]. Although Id2 determines the commitment and maintenance of the entire NK/ILC lineage, *Rroid*^−/−^ mice have no defects in common helper ILC progenitors and in other ILC subsets, implying that specific regulatory elements control Id2 transcription during different developmental stages of ILCs. In particular, for NK cells and ILC1, these regulatory mechanisms are regulated by IL-15. At a mechanistic level, the *Rroid* locus, but not lncRNA itself, is required for IL-15/STAT5 mediated-activation of Id2 promoter. The *Rroid* locus and the *Id2* promoter are adjacent and can form a long-range loop which renders chromatin properly accessible to favor the binding of STAT5 to Id2 promoter.

The lncKdm2b, instead, is specifically highly expressed in ILC3 and plays a key regulatory function in these cells. Accordingly, two different mouse models, established to delete *lncKdm2b* in the hematopoietic system or only in ILC3, revealed selective effects of lncKdm2b on this subset, with a strong decrease in the absolute number and effector functions. These effects are due to the capability of lncKdm2b to control ILC3 proliferation, and the regulation of the expression of the TF Zfp929 has an important role in this mechanism. At a molecular level, lncKdm2b binds Satb1, a genome-organizer protein able to recruit the chromatin-remodeling complex NURF to *Zfp929* promoter and to trigger its transcription [95].

## 4. Regulation of ILC Activity by circRNAs

### 4.1. Properties of circRNAs

circRNAs represent a category of nc-RNAs characterized by a continuous RNA sequence without open 3′ and 5′ end. Thanks to their covalent closed-loop structure, circRNAs are protected from degradation by RNases, thus displaying a higher stability than linear RNAs [96,97]. For decades, circRNAs have been considered as the anomalous products of splicing, but recent advances in high-throughput RNA sequencing have unveiled new information about their functions. There are four main subtypes of circRNAs: exonic circRNAs (ecircRNAs), mainly characterized by a single or several exons; circular intronic RNAs (ciRNAs), containing only introns; exonic–intronic circRNAs (EIciRNAs), including both introns and exons; and tRNA intronic circRNAs (tricRNAs), formed by the splicing of pre-tRNA intron. Most of the circRNAs are composed of single or multiple exons [98], and their expression is developmentally regulated and tissue and cell-type specific [99]. CircRNAs are produced by a lariat-driven circularization or back-splicing, a process that occurs in a reversed orientation as compared with canonical splicing [98]. MiRNA sponge activity is the most frequently described function of circRNAs. They interact with miRNAs by preventing their inhibitory activity on canonical mRNA targets. Other annotated functions include the sponging of proteins, scaffolds for protein complex, modulation of transcription, and splicing [100,101]. Recent studies indicated that some cytoplasmic circRNAs can be also translated into regulatory peptides. Thus, these circRNAs can exert their biological functions both through encoded peptides and/or by RNA-based regulatory mechanisms. In particular, circRNA-translated proteins play pivotal roles in cancer by promoting/inhibiting tumorigenesis [101,102].

### 4.2. circRNAs and ILCs

The immunoregulatory properties of circRNAs are now starting to be understood [103]. circRNAs have been implicated in immune responses against microbial infections and cancer. Recent studies have demonstrated the critical functions of circRNAs in NK cells and ILC3 (Figure 1, lower panel). They can regulate the antitumor NK cell activity [104]. In both tumor tissues and plasma exosomal RNA of patients with hepatocarcinoma (HCC), the expression of the UHRF1-derived circular RNA, named circUHRF1, circUHRF1 is increased and is associated with decreased NK cell proportion and tumor infiltration. Exosomal circUHRF1 secreted by HCC cells can be delivered into NK cells, by inducing the expression of the inhibitory receptor TIM-3 and inhibiting IFN-γ and TNF-α production. At the molecular level, a peculiar regulatory circuit connects this circRNA with a miRNA able to target *TIM-3* mRNA, the miR-449c-5p. The circUHRF1 acts as a binding platform for miR-449c-5p and inhibits its activity, thus promoting the expression of TIM-3 in NK cells. The relevance of this circRNA in mediating NK cell dysfunction in liver cancer has been highlighted by observations on its role in anticancer therapy. In a mouse xenograft model, the subcutaneous implantation of circUHRF1-knockdown HCCLM3 cells resulted in sensitivity to anti-PD1 treatment and in increasing in the overall survival rate; consistently, a retrospective study on a cohort of 30 HCC patients treated with anti-PD1 mAb suggested that high levels of tumor circUHRF1 positively correlate with progressive disease. These findings suggest the possibility to use this circRNA both as a prognostic biomarker as well as a therapeutic target.

In the context of intestinal inflammation, circZbtb20 and circKcnt2 exert relevant effects on ILC3 activity. *CircZbtb20* knockout mice show a reduced percentage and number of intestinal ILC3, also defective in IL-22 production, and increased the susceptibility to *C. rodentium* infection. Such effects can be attributed to the alteration of the Notch pathway required for ILC3 proliferation and functions [105]. Mechanistically, upon interaction with Nr4a1 mRNA, CircZbtb20 recruits the Alkbh5 demethylase to remove the m6A modification responsible for its stability. Thus, the CircZbtb20 promotes the expression of transcription factor Nr4a by enhancing the stability of its mRNA. Then, Nr4a1 directs the expression of genes correlated to the Notch signaling pathway, such as Notch2.

While CircZbtb20 is constitutively present in intestinal ILC3, circKcnt2 transcription is activated only in colitis-associated ILC3. Mice lacking *circKcnt2* displayed much more innate colitis and more IL-17 production by ILC3 [106]. A transcriptome analysis of ILC3 *circKcnt2*^−/−^ vs. *circKcnt2*^+/+^ contributed to elucidating the molecular mechanisms of circKcnt2 in the promotion of colitis, by revealing Batf as the most upregulated TF in the absence of the circRNA. The circKcnt2 recruits a transcriptional repressor, the NuRD complex on *Batf* promoter, and suppresses its transcription also leading to the inhibition of IL-17a expression, one of target genes of this transcription factor.

## 5. Conclusions

It is now clear that ncRNAs can control the gene expression by generating fine-tuned regulatory circuits. Recent advances in next-generation sequencing techniques and bioinformatics approaches have enabled the profiling of miRNAs, lncRNAs, and circRNAs in a large variety of cells and have elucidated their role in diverse biological processes. Tight control mechanisms guarantee the concerted action of multiple ncRNAs generating complex regulatory RNA networks also strictly interconnected with many other regulatory elements.

The contribution of these regulatory circuits to the molecular programs required for the development and functions of ILCs is also emerging (Table 1). However, our knowledge in this field is still limited and puzzling. While the role of miRNAs in NK cell biology has been investigated, how they operate in other ILC subsets remains to be elucidated. Genetic approaches in mice have led to the identification of specific functions of miRNAs in ILCs. Interestingly, the shared expression of discrete groups of miRNAs among ILCs opens the possibility that these molecules could help determine innate vs. adaptive signatures. Differently, the specific patterns of expression of miRNAs can account for the peculiarities of distinct ILC subpopulations. Comprehensive comparisons of miRNome among ILC subsets and between ILCs and Th cell counterparts would be helpful for understanding whether and how these regulatory RNAs concur to generating the heterogeneity of these lymphocytes. Similar approaches should be also used to profile lnc- and circRNAs in these immune cells. Despite the limited information on lncRNAs and circRNAs in ILCs, the evidence encourages further investigation of their pattern of expression and regulatory functions; it is plausible that also these ncRNAs are crucial for the imprinting of ILC identity and functions. A further level of complexity comes from difficulties in translating mouse studies to humans, due to the limited conservation of ncRNAs among species and to the phenotypical and functional differences between human and mouse ILCs. Additional studies might provide further insight into the roles of ncRNAs in ILCs.

To date, a role for ncRNAs on ILC plasticity has not been demonstrated. However, several studies reported the regulation of these transcripts by cytokines, which are critical factors to driving the behavior and function of ILCs [107], thus suggesting the involvement of ncRNAs in these mechanisms. Although still challenging from a technical point of view, it will be highly important to profile ncRNAs in immune cells at single cell resolution, both in homeostatic and pathological conditions. Indeed, beyond the importance of deconvoluting ncRNA-dependent regulatory circuits, this information is particularly relevant in the design of therapeutic approaches based on ncRNA delivery.

## Figures and Tables

**Table 1:** Functional ncRNAs in ILCs.

ncRNAs	Cell	Regulator	Target	Biological Effect	References
**miRNAs**					
miRNA-142-3p	ILC1	IL-15	TGFBR1	↓ TGFβ signalling	[58]
miRNA-142-5p	ILC1	IL-15	SOCS1	↑ IL-15 signalling	[58]
miRNA-142	ILC2	-	SOCS1	↑ γc-cytokine signalling	[62]
miRNA-142	ILC2	-	GFI1	↓ ST2-IL-33 signalling	[62]
miRNA-19a	ILC2	-	SOCS1	↑ JAK/STAT signalling	[63]
miRNA-19a	ILC2	-	TNFAIP3	↑ IL-13 and IL-5 signalling	[63]
miRNA-155	ILC2	IL-33	c-Maf	↓ IL-4, IL-5, IL-9 and IL-13 production	[11,69]
miRNA-146a	ILC2	-	TRAF6, IRAK1	↓ ST2-IL-33 signalling	[71]
**lncRNAs**					
lnc-CD56	NK	-	CD56	↑ NK cell differentiation	[86]
lnc-GAS5	NK	IL-2	RUNX3	↑ NK cell cytotoxicity	[91]
lnc-ifng-as	NK	STAT-4/ T-BET, IL-12/IL-18	IFN-γ	↑ IFN-γ production	[84,85]
Rroid locus	ILC1	IL-15	Id2	↓ T and B cell lineage	[92]
lncKdm2b	ILC3	-	Zfp929	↑ ILC3 proliferation	[95]
**circRNAs**					
circUHRF1	NK	Tumor	TIM-3	↓ IFN-γ and TNF-α production	[104]
circZbtb20	ILC3	-	Nr4a	↑ ILC3 proliferation	[105]
circKcnt2	ILC3	Inflammation	Batf	↓ IL-17a expression and ILC3 activation	[106]

↑: Increase; ↓: Decrease; - Not determined.

## Data Availability

Not applicable.

## Abbreviations

ILC	innate lymphoid cell
TF	transcription factor
NK	natural killer
ILC1	type-1 innate lymphoid cell
IFN	interferon
TGF-β	transforming growth factor-β
ILC2	type-2 innate lymphoid cell
IL	interleukin
ILC3	type-3 innate lymphoid cell
LTi	lymphoid tissue inducer
LDTF	lineage defining TF
ncRNA	noncoding RNA
miRNA	microRNA
rRNA	ribosomal RNA
tRNA	transfer RNA
lncRNA	long ncRNA
circRNA	circular RNA
RISC	RNA-induced silencing complex
H3K27me3	trimethylation of lysine 27 of the histone 3
ILCp	ILC precursor
a-LP	a-lymphoid progenitors
dILC3	decidual ILC3
dNK	decidual NK
pbNK	peripheral blood NK cells
cbNK	cord blood NK
ecircRNAs	exonic circRNAs
ciRNAs	circular intronic RNAs
EIciRNAs	exonic–intronic circRNAs
tricRNAs	tRNA intronic circRNAs.
